# Influence of cytokines on the recovery trajectory of HIV patients on antiretroviral therapy: A review

**DOI:** 10.1097/MD.0000000000041222

**Published:** 2025-01-03

**Authors:** Emmanuel Ifeanyi Obeagu

**Affiliations:** a Department of Medical Laboratory Science, Kampala International, University, Ishaka Uganda.

**Keywords:** antiretroviral therapy, cytokines, HIV, inflammation

## Abstract

Cytokines, critical signaling molecules in the immune system, significantly influence the pathophysiology of Human Immunodeficiency Virus (HIV) infection and the effectiveness of antiretroviral therapy (ART). Dysregulated cytokine production, characterized by elevated pro-inflammatory and anti-inflammatory cytokines, plays a pivotal role in chronic inflammation and immune activation in untreated HIV patients. ART initiation leads to changes in cytokine levels, typically resulting in decreased systemic inflammation, though the extent and persistence of these changes vary among individuals. Despite successful viral suppression with ART, many HIV patients experience persistent immune activation and inflammation, driven by ongoing cytokine dysregulation. This persistent inflammatory state is associated with adverse clinical outcomes, including cardiovascular disease, neurocognitive impairment, and non-AIDS-related cancers. Understanding the specific cytokine profiles that contribute to these outcomes is crucial for developing targeted therapeutic interventions to improve long-term health. Cytokine modulation presents a promising avenue for enhancing immune recovery and reducing chronic inflammation in HIV patients on ART. Identifying cytokine patterns that serve as biomarkers for disease progression and treatment response can help tailor individualized treatment strategies. Future research should focus on adjunctive therapies that target cytokine activity to mitigate residual inflammation, thereby improving the overall health and quality of life for HIV patients.

## 1. Introduction

Human Immunodeficiency Virus (HIV) remains 1 of the most significant global health challenges, despite advances in medical research and treatment. The advent of antiretroviral therapy (ART) has transformed HIV from a fatal disease to a chronic manageable condition, enabling millions of people living with HIV to lead healthier and longer lives. ART works by suppressing the viral load to undetectable levels, thereby preventing the progression of HIV to acquired immunodeficiency syndrome (AIDS) and reducing the risk of HIV transmission. However, the recovery trajectory of HIV patients on ART is influenced by various factors, including the critical role of cytokines.^[1,2]^ Cytokines are small proteins that are crucial for cell signaling in the immune system. They are produced by a broad range of cells, including immune cells like macrophages, B lymphocytes, T lymphocytes, and mast cells. Cytokines play diverse roles in mediating and regulating immunity, inflammation, and hematopoiesis. In the context of HIV infection, cytokines are instrumental in both the immune response to the virus and the pathological processes that drive disease progression. The balance between pro-inflammatory and anti-inflammatory cytokines is particularly significant in determining the extent of immune activation and inflammation, which are key factors in HIV pathogenesis.^[3,4]^ Before the initiation of ART, HIV infection is characterized by marked dysregulation of cytokine production. Pro-inflammatory cytokines such as tumor necrosis factor-alpha (TNF-α), interleukin-1 beta (IL-1β), and interleukin-6 (IL-6) are typically elevated in untreated HIV patients. This heightened state of inflammation contributes to chronic immune activation, which is a hallmark of HIV infection and a major driver of CD4^+^ T cell depletion. Chronic immune activation leads to a vicious cycle of immune system exhaustion and further immune suppression, exacerbating the progression of HIV disease.^[5]^ The introduction of ART brings about significant changes in cytokine profiles. ART reduces the viral load, which subsequently decreases the levels of pro-inflammatory cytokines and overall systemic inflammation. This reduction in inflammation is a positive outcome, as it is associated with decreased immune activation and improved immune function. However, the degree to which cytokine levels normalize varies among individuals, and some patients continue to exhibit signs of chronic inflammation despite effective viral suppression. This persistent inflammation is a critical area of concern as it can influence the long-term health outcomes of HIV patients.^[6]^

CD4^+^ T cell recovery is a primary goal of ART, and cytokines play a vital role in this process. Interleukin-2 (IL-2) is essential for the proliferation and survival of T cells, and its levels are often correlated with better immune recovery. Conversely, elevated levels of cytokines such as IL-6 and TNF-α may hinder optimal immune reconstitution by promoting continuous immune activation and apoptosis of T cells. The interplay between different cytokines and their effects on CD4^+^ T cell recovery highlights the complexity of immune reconstitution in HIV patients on ART.^[7]^ Despite effective ART, many HIV patients experience persistent immune activation and inflammation. This ongoing inflammatory state is driven by several factors, including microbial translocation, co-infections, and residual viral replication. Elevated levels of pro-inflammatory cytokines like IL-6, IL-1β, and TNF-α are associated with an increased risk of non-AIDS-related comorbidities such as cardiovascular diseases, neurocognitive disorders, and certain cancers. These conditions significantly impact the quality of life and overall prognosis of HIV patients, underscoring the need for comprehensive management strategies that address both viral suppression and inflammation.^[8,9]^ Chronic inflammation and immune activation pose significant challenges in the management of HIV, even in the era of effective ART. Persistent cytokine dysregulation is a major contributor to these challenges, and it necessitates a deeper understanding of the underlying mechanisms. Research into the modulation of cytokine activity offers promising avenues for improving the long-term health outcomes of HIV patients. Anti-inflammatory agents, cytokine inhibitors, and other adjunctive therapies are being explored to reduce residual inflammation and enhance immune recovery.^[10]^ Cytokine profiles can also serve as valuable biomarkers for monitoring disease progression and treatment response in HIV patients. Identifying specific cytokine patterns that are associated with poor outcomes can help in tailoring individualized treatment strategies. For example, patients with persistently high levels of certain pro-inflammatory cytokines may benefit from targeted therapies that specifically address these inflammatory pathways. This personalized approach to HIV management could lead to more effective treatments and better health outcomes.^[11]^

### 1.1. Aim

The aim of this review article is to comprehensively explore the influence of cytokines on the recovery trajectory of HIV patients receiving ART.

### 1.2. Rationale

Despite the success of ART in suppressing HIV replication and prolonging life expectancy, HIV-infected individuals continue to face significant health challenges associated with chronic inflammation and immune dysfunction. These challenges manifest as increased risks of cardiovascular disease, neurocognitive disorders, metabolic abnormalities, and other non-AIDS-related conditions. Cytokines play a pivotal role in orchestrating immune responses and inflammatory processes. In HIV infection, cytokine dysregulation contributes to persistent immune activation, even in the presence of effective viral suppression with ART. Understanding how cytokines influence immune recovery and contribute to chronic inflammation is essential for developing targeted therapies to mitigate these effects. The levels of cytokines such as IL-6, TNF-α, and others serve as biomarkers of inflammation and predictors of disease progression in HIV patients. Monitoring cytokine profiles can provide insights into treatment efficacy, immune reconstitution, and the likelihood of developing comorbidities. Insights gained from this review can inform clinical practice by guiding the selection of biomarkers for monitoring HIV progression and response to therapy. Moreover, understanding the mechanisms through which cytokines influence CD4^+^ T cell recovery can lead to the development of novel therapeutic strategies aimed at optimizing immune restoration and reducing inflammation-related complications. By synthesizing current knowledge on cytokine dynamics in HIV pathogenesis and treatment, this review aims to identify gaps in understanding and propose avenues for future research. These may include exploring new biomarkers, investigating cytokine-targeted therapies, and conducting longitudinal studies to assess long-term outcomes in HIV patients.

## 2. Review methodology

### 2.1. Search strategy

*Literature search:* A systematic search was conducted using electronic databases including PubMed, Google Scholar, and Scopus. The search terms used included combinations of keywords such as “HIV,” “cytokines,” “antiretroviral therapy,” “immune activation,” “inflammation,” “CD4^+^ T cell recovery,” “biomarkers,” and “therapeutic targets.”

*Inclusion criteria:* Studies were included if they focused on cytokine profiles in HIV-infected individuals, particularly those on ART. Both clinical trials and observational studies were considered. Articles written in English and published within the last 10 years were prioritized, with older seminal works included for foundational understanding.

*Exclusion criteria:* Studies focusing exclusively on non-HIV-related conditions, animal studies, reviews without primary data, and studies lacking relevance to cytokine dynamics in HIV were excluded.

### 2.2. Data collection and analysis

*Screening and selection:* Titles and abstracts of identified articles were screened for relevance to the review objectives. Full texts of potentially relevant articles were then reviewed to determine eligibility based on inclusion criteria.

*Data extraction:* Data extraction focused on key elements including study design, participant characteristics (e.g., HIV status, ART regimen), cytokine measurements, outcomes related to immune activation and inflammation, and implications for clinical management.

*Synthesis of results:* Synthesis involved categorizing findings into thematic sections such as pre-ART cytokine dysregulation, changes in cytokine profiles with ART initiation, mechanisms of CD4^+^ T cell recovery, persistent immune activation and inflammation, biomarkers for monitoring, and potential therapeutic targets.

## 3. Cytokine profiles in HIV infection

### 3.1. Pre-ART cytokine dysregulation

In the context of untreated HIV infection, cytokine dysregulation is a critical factor that contributes to disease progression. Cytokines, which are small signaling proteins involved in immune responses, become imbalanced, leading to a state of chronic immune activation and inflammation. This dysregulation is characterized by elevated levels of pro-inflammatory cytokines and, in some cases, compensatory increases in anti-inflammatory cytokines. The imbalance and chronic activation play a central role in the pathogenesis of HIV, driving both immune system damage and the progression towards acquired immunodeficiency syndrome (AIDS).^[12]^ Pro-inflammatory cytokines such as TNF-α, IL-1β, and IL-6 are markedly elevated in individuals with untreated HIV infection. TNF-α, a key mediator of inflammation, is produced by various immune cells, including macrophages and T cells, in response to HIV infection. Its overproduction leads to a cascade of inflammatory responses that contribute to the destruction of CD4^+^ T cells, a hallmark of HIV progression. IL-1β and IL-6, similarly elevated, further enhance inflammatory pathways and immune activation, exacerbating the immune system’s dysfunction.^[12]^ While pro-inflammatory cytokines dominate the cytokine milieu in untreated HIV, there is also an upregulation of certain anti-inflammatory cytokines, such as interleukin-10 (IL-10). IL-10 is produced by a variety of immune cells, including T regulatory cells and macrophages, and functions to limit excessive immune activation and inflammation. However, in the context of HIV infection, the increase in IL-10 may not be sufficient to counterbalance the overwhelming pro-inflammatory environment. Moreover, elevated IL-10 can contribute to immune exhaustion by inhibiting the function of CD4^+^ and CD8^+^ T cells, further impairing the immune response against HIV.^[13]^ The mechanisms driving cytokine dysregulation in untreated HIV infection are multifaceted. Direct infection of immune cells by HIV leads to the activation of these cells and the subsequent release of cytokines. Additionally, HIV proteins such as gp120 and Tat have been shown to directly stimulate cytokine production. Another significant factor is microbial translocation, where the integrity of the gut mucosa is compromised, allowing microbial products to enter the bloodstream. These microbial products act as potent stimuli for the production of pro-inflammatory cytokines, thereby perpetuating immune activation and inflammation.^[13]^

The chronic elevation of pro-inflammatory cytokines has several detrimental effects on the immune system. It leads to the persistent activation and turnover of CD4^+^ T cells, accelerating their depletion. This continuous immune activation also promotes the apoptosis of immune cells, further contributing to immune system decline. Additionally, the inflammatory environment favors the expansion of immune-suppressive cells, such as T regulatory cells, which inhibit effective immune responses against HIV. These processes collectively result in a weakened immune system, reduced ability to control opportunistic infections, and increased progression to AIDS.^[14]^ Elevated cytokine levels are associated not only with immune system damage but also with increased risk of comorbidities such as cardiovascular disease and neurocognitive disorders. This highlights the need for interventions that can modulate cytokine levels and reduce chronic inflammation. While ART significantly reduces viral load and systemic inflammation, adjunctive therapies targeting specific cytokines or their signaling pathways may further enhance immune recovery and reduce the risk of long-term complications.^[14]^

### 3.2. Cytokine changes with ART initiation

The initiation of ART in HIV-infected individuals has a profound impact on cytokine profiles. ART effectively suppresses HIV replication, leading to a rapid decline in plasma viral load. This viral suppression is associated with a significant reduction in systemic inflammation and immune activation, reflected by changes in cytokine levels. Pro-inflammatory cytokines, such as TNF-α, IL-1β, and IL-6, typically decrease shortly after the initiation of ART. This reduction in pro-inflammatory cytokines indicates a dampening of the chronic inflammatory state induced by ongoing HIV replication.^[15]^ Over the course of ART, cytokine levels continue to evolve, reflecting the dynamic process of immune system recovery. Studies have shown that while there is an initial sharp decline in pro-inflammatory cytokines, the normalization of cytokine levels can vary among individuals and may take several months to years. Anti-inflammatory cytokines, such as IL-10, may also decrease, although this change is often less pronounced than that of pro-inflammatory cytokines. The longitudinal monitoring of cytokine profiles provides insights into the extent and quality of immune recovery under ART.^[16]^ Several factors influence the degree and rate of cytokine normalization following ART initiation. The timing of ART initiation is critical; earlier initiation of ART, often during the acute phase of HIV infection, is associated with more pronounced and rapid reductions in inflammatory cytokines. The specific antiretroviral regimen can also impact cytokine levels, with some drugs potentially having more potent anti-inflammatory effects than others. Additionally, individual patient characteristics, such as age, co-infections, and the presence of comorbid conditions, can modulate the cytokine response to ART.

### 3.3. Persistent immune activation despite ART

Despite the overall trend towards decreased cytokine levels with ART, a subset of patients continues to exhibit persistent immune activation and elevated inflammatory cytokines. This phenomenon, often referred to as “residual inflammation,” can persist even in the context of sustained viral suppression. Factors contributing to persistent immune activation include microbial translocation, co-infections (such as cytomegalovirus or hepatitis C), and residual HIV replication in sanctuary sites. Elevated levels of cytokines like IL-6 and TNF-α in these patients are associated with an increased risk of non-AIDS-related comorbidities, including cardiovascular disease and neurocognitive disorders.^[17]^ The modulation of cytokine levels by ART has direct implications for CD4^+^ T cell recovery, a key indicator of immune system restoration in HIV patients. Lower levels of pro-inflammatory cytokines post-ART are generally associated with better CD4^+^ T cell recovery. Cytokines such as IL-2, crucial for T cell proliferation and survival, play a significant role in this recovery process. However, persistent elevation of inflammatory cytokines can impair CD4^+^ T cell reconstitution by promoting apoptosis and immune exhaustion.^[16]^ To address persistent inflammation and optimize immune recovery, adjunctive therapeutic strategies targeting specific cytokines or their signaling pathways are being explored. Anti-inflammatory agents, such as statins and nonsteroidal anti-inflammatory drugs (NSAIDs), and cytokine inhibitors like monoclonal antibodies against IL-6 or TNF-α, have shown potential in reducing residual inflammation. These interventions aim to complement ART by further reducing cytokine-driven immune activation and improving overall health outcomes.^[17]^ Cytokine levels can serve as valuable biomarkers for monitoring treatment response and guiding therapeutic decisions in HIV patients on ART. Regular assessment of cytokine profiles can help identify individuals at risk for persistent inflammation and associated comorbidities. Personalized treatment approaches, informed by cytokine biomarkers, can enhance the efficacy of ART and adjunctive therapies, leading to better long-term management of HIV.

### 3.4. Cytokines and immunological response in HIV

In the context of HIV infection, cytokines play a pivotal role in orchestrating and regulating the immune response.^[12]^ The immune system’s complex network of cytokines – small signaling proteins produced by various cells – serves as a fundamental component in the body’s defense against pathogens, including HIV.^[13]^ When HIV infects a host, it primarily targets CD4^+^ T cells, which are central to coordinating immune responses.^[14]^ The virus uses these cells as a replication site, ultimately leading to their destruction. This destruction, coupled with the chronic activation of the immune system in response to the persistent viral infection, results in dysregulated cytokine production and subsequent immunological alterations. Cytokines can be broadly categorized into different groups based on their functions and roles in the immune response.^[15]^ Some cytokines, such as interleukins, interferons (IFNs), tumor necrosis factors, and chemokines, regulate immune cell proliferation, differentiation, and activation.^[16]^ They also influence the communication and recruitment of immune cells to sites of infection or inflammation.

In HIV infection, there is a dysregulation of cytokine production and activity.^[17]^ Elevated levels of pro-inflammatory cytokines, such as IL-1, IL-6, and TNF-α, contribute to chronic immune activation and inflammation, which can lead to immune system exhaustion and tissue damage.^[18]^ Concurrently, there might be a decrease in certain cytokines that are crucial for immune regulation and function, like IL-2 and IFN-gamma, impairing the body’s ability to mount an effective antiviral response.^[19]^ Furthermore, cytokines play a crucial role in the HIV life cycle. Some cytokines, like certain chemokines (e.g., RANTES, MIP-1α, and MIP-1β), have the ability to inhibit HIV replication by interfering with viral entry or by blocking viral transcription.^[20]^ Conversely, HIV itself can manipulate cytokine networks, promoting its replication and evading immune responses. Understanding the intricate balance and interactions between cytokines during HIV infection is crucial. These cytokine dynamics influence the progression of the disease, the effectiveness of ART, and the overall immune reconstitution of HIV patients.^[21]^ Researchers are investigating ways to modulate cytokine responses to enhance antiviral immunity and minimize immune activation, aiming to improve treatment outcomes and mitigate HIV-associated complications. Cytokines play a multifaceted role in the immunological response to HIV infection.^[22]^ Dysregulation of cytokine networks contributes significantly to immune dysfunction and disease progression in HIV-infected individuals, highlighting the importance of exploring cytokine modulation as a potential therapeutic avenue in managing HIV/AIDS.^[23]^

## 4. Impact of cytokines on immune recovery

### 4.1. CD4^+^ T cell recovery

CD4^+^ T cells play a critical role in the immune response by aiding in the activation and regulation of other immune cells. HIV infection targets and depletes these cells, leading to immune system dysfunction and progression to AIDS. The primary goal of ART is to suppress HIV replication, which allows the immune system to recover, as indicated by the restoration of CD4^+^ T cell counts. Successful CD4^+^ T cell recovery is associated with improved immune function, reduced risk of opportunistic infections, and overall better health outcomes. HIV primarily infects CD4^+^ T cells, leading to their direct destruction. Additionally, chronic immune activation and inflammation contribute to CD4^+^ T cell depletion. Pro-inflammatory cytokines like TNF-α and IL-6 promote apoptosis and immune exhaustion, further reducing CD4^+^ T cell numbers. The persistent immune activation observed in untreated HIV infection drives the continuous turnover and depletion of these critical cells. Initiation of ART leads to a significant reduction in viral load, which is closely followed by a decrease in systemic inflammation and immune activation. This creates a more favorable environment for the recovery of CD4^+^ T cells. Typically, there is a rapid initial increase in CD4^+^ T cell counts during the first few months of ART, primarily due to the redistribution of memory T cells from lymphoid tissues into the bloodstream. This is followed by a slower, sustained increase over the ensuing years, reflecting true regeneration of the CD4^+^ T cell pool through thymic output and peripheral proliferation. Patients who initiate ART with higher baseline CD4^+^ T cell counts generally experience better recovery compared to those who start treatment with severe immunosuppression. Younger individuals tend to have more robust thymic function, leading to better regeneration of CD4^+^ T cells compared to older patients. The presence of other infections (e.g., hepatitis B or C, cytomegalovirus) and chronic conditions can impair immune recovery by maintaining a state of immune activation and inflammation. Early initiation of ART, often during the acute phase of infection, is associated with more favorable CD4^+^ T cell recovery and overall better long-term outcomes.^[17–19]^

### 4.2. Role of cytokines in CD4^+^ T cell recovery

Cytokines play a dual role in influencing CD4^+^ T cell recovery. Certain cytokines, such as IL-2, are crucial for T cell proliferation and survival. ART-induced reduction in pro-inflammatory cytokines (e.g., TNF-α, IL-6) alleviates the apoptotic and inhibitory signals that hinder CD4^+^ T cell regeneration. However, persistent elevation of these pro-inflammatory cytokines in some patients can continue to impair CD4^+^ T cell recovery despite effective viral suppression. Despite the overall benefits of ART, a subset of patients experiences incomplete CD4^+^ T cell recovery. These individuals often exhibit signs of ongoing immune activation and elevated inflammatory markers. Persistent immune activation can be driven by factors such as microbial translocation, co-infections, and residual viral replication in sanctuary sites. Incomplete CD4^+^ T cell recovery is associated with an increased risk of non-AIDS-related complications, including cardiovascular disease and neurocognitive disorders. Drugs such as statins and NSAIDs have shown potential in reducing chronic inflammation and promoting CD4^+^ T cell recovery. Therapies targeting specific cytokines or their receptors, such as monoclonal antibodies against IL-6 or TNF-α, aim to reduce immune activation and support immune restoration. Strategies such as IL-2 therapy, designed to boost T cell proliferation, and therapies aimed at enhancing thymic function are being investigated for their potential to improve CD4^+^ T cell recovery. Monitoring cytokine levels and immune activation markers can provide valuable insights into the efficacy of ART and the likelihood of CD4^+^ T cell recovery. Biomarkers such as IL-6, TNF-α, and soluble CD14 can help identify patients at risk for incomplete immune recovery and guide the implementation of adjunctive therapies.^[18–20]^

## 5. Persistent immune activation and inflammation

Despite the effectiveness of ART in suppressing HIV replication, a subset of patients continues to experience persistent immune activation and inflammation. This ongoing inflammatory state poses significant challenges to achieving optimal immune recovery and contributes to various comorbidities, including cardiovascular disease, neurocognitive disorders, and non-AIDS-related cancers. Understanding the mechanisms driving persistent immune activation is crucial for developing interventions to mitigate its effects and improve long-term health outcomes for HIV patients. Damage to the gut mucosa, a common consequence of HIV infection, allows microbial products such as lipopolysaccharides to translocate into the bloodstream. These microbial products act as potent stimuli for the innate immune system, perpetuating systemic inflammation. Even with effective ART, low-level HIV replication can occur in sanctuary sites where the virus remains protected from the effects of the drugs. This residual viral activity can continuously stimulate the immune system. Co-infections with other pathogens, such as hepatitis B or C, cytomegalovirus, and tuberculosis, can maintain a state of immune activation. These infections often require additional immune resources and can exacerbate the inflammatory response. Chronic HIV infection leads to immune senescence, characterized by the accumulation of aged and exhausted T cells. These cells exhibit altered cytokine production and reduced proliferative capacity, contributing to ongoing immune activation.^[21]^

## 6. Cytokine profiles in persistent inflammation

Persistent immune activation is marked by sustained elevation of pro-inflammatory cytokines such as IL-6, TNF-α, and IL-1β. Elevated IL-6 levels are linked to increased cardiovascular risk, as this cytokine promotes the development of atherosclerosis and other vascular complications. IL-6 also plays a role in the activation of the acute-phase response, which can contribute to systemic inflammation. TNF-α is a central mediator of inflammation and is involved in the pathogenesis of several inflammatory diseases. In HIV patients, elevated TNF-α levels are associated with immune activation, apoptosis of CD4^+^ T cells, and the maintenance of viral reservoirs. This cytokine contributes to chronic inflammation and is implicated in the development of neurocognitive disorders in HIV patients. IL-1β promotes the activation of microglia and astrocytes in the brain, leading to neuro-inflammation and potential neurodegeneration. Chronic inflammation contributes to the development of atherosclerosis and other cardiovascular conditions. HIV patients with elevated inflammatory markers have a higher risk of myocardial infarction, stroke, and other cardiovascular events. Persistent inflammation is associated with HIV-associated neurocognitive disorders (HAND), which range from asymptomatic neurocognitive impairment to severe dementia. Inflammatory cytokines like IL-1β and TNF-α play critical roles in neuro-inflammation and neuronal damage. Chronic immune activation and inflammation increase the risk of developing non-AIDS-related cancers. Elevated cytokine levels create a pro-tumorigenic environment by promoting cellular proliferation, inhibiting apoptosis, and inducing angiogenesis.^[21,22]^

Medications such as statins, NSAIDs, and corticosteroids are being investigated for their potential to reduce systemic inflammation in HIV patients. These agents may help lower the levels of pro-inflammatory cytokines and improve immune function. Targeted therapies that block specific cytokines or their receptors, such as monoclonal antibodies against IL-6 or TNF-α, have shown promise in reducing inflammation. These inhibitors can help mitigate the effects of persistent immune activation and improve clinical outcomes. Strategies to restore gut integrity and reduce microbial translocation, such as probiotics, prebiotics, and gut-specific anti-inflammatory agents, are being explored. Improving gut health may help lower systemic inflammation and enhance immune recovery. Managing co-infections effectively can reduce the burden on the immune system and decrease overall inflammation. Early detection and treatment of co-infections are crucial for minimizing their impact on immune activation. Identifying and monitoring biomarkers of inflammation can help guide the management of HIV patients on ART. Biomarkers such as IL-6, TNF-α, C-reactive protein (CRP), and soluble CD14 (sCD14) are useful indicators of systemic inflammation and immune activation. Regular assessment of these biomarkers can aid in identifying patients at risk for complications and tailoring individualized treatment strategies.^[23]^

## 7. Long-term effects of cytokine modulation

### 7.1. Chronic inflammation and comorbidities

Chronic inflammation is a hallmark of HIV infection, persisting even in patients who are on effective ART. This sustained inflammatory state contributes significantly to the development of various comorbidities that affect the long-term health and quality of life of HIV-infected individuals. Understanding the link between chronic inflammation and these comorbid conditions is crucial for developing comprehensive management strategies for HIV patients. Despite effective ART, low-level HIV replication can persist in sanctuary sites, continuously stimulating the immune system. Damage to the gut mucosa from HIV infection allows bacterial products, such as lipopolysaccharides, to enter the bloodstream, triggering systemic inflammation. Concurrent infections with other pathogens, such as hepatitis B and C, cytomegalovirus, and tuberculosis, can maintain a state of immune activation. Chronic HIV infection leads to immune senescence, characterized by the accumulation of aged and exhausted T cells, which produce inflammatory cytokines and contribute to the inflammatory milieu.^[17]^

Chronic inflammation significantly increases the risk of cardiovascular disease in HIV-infected individuals. Elevated levels of pro-inflammatory cytokines such as IL-6 and TNF-α are associated with endothelial dysfunction, atherosclerosis, and plaque formation. HIV patients are at a higher risk for myocardial infarction, stroke, and other cardiovascular events compared to the general population. ART, while reducing viral load and systemic inflammation, does not completely eliminate this risk, highlighting the need for additional interventions to manage cardiovascular health in HIV patients. HAND encompass a spectrum of cognitive impairments ranging from mild neurocognitive disorder to severe dementia. Chronic inflammation plays a pivotal role in the pathogenesis of HAND. Pro-inflammatory cytokines such as IL-1β and TNF-α contribute to neuro-inflammation, leading to neuronal damage and cognitive decline. Persistent immune activation in the central nervous system can also promote the activation of microglia and astrocytes, further exacerbating neurodegeneration. Managing chronic inflammation is essential for preventing and treating neurocognitive impairments in HIV patients.^[18]^

HIV-infected individuals are at an increased risk of developing metabolic syndrome and diabetes, conditions characterized by a cluster of metabolic abnormalities including insulin resistance, dyslipidemia, and hypertension. Chronic inflammation is a key driver of these metabolic disturbances. Elevated levels of inflammatory markers such as CRP and IL-6 are linked to insulin resistance and impaired glucose metabolism. ART regimens, particularly those including protease inhibitors, can exacerbate metabolic issues, necessitating careful monitoring and management of metabolic health in HIV patients. Bone disorders, including osteoporosis and osteopenia, are more prevalent in HIV-infected individuals. Chronic inflammation contributes to bone resorption and decreased bone mineral density. Inflammatory cytokines such as TNF-α and IL-6 promote osteoclast activity, leading to increased bone turnover and reduced bone strength. ART, while beneficial for viral suppression, can also impact bone health, with some antiretroviral drugs being associated with bone density loss. Addressing inflammation and ensuring adequate calcium and vitamin D intake are important for maintaining bone health in HIV patients. Renal disease is a significant comorbidity in HIV patients, with chronic inflammation playing a major role in its development. Inflammatory cytokines contribute to glomerular and tubular damage, leading to conditions such as HIV-associated nephropathy and chronic kidney disease. ART has improved renal outcomes for many patients, but the persistence of inflammation necessitates ongoing monitoring of kidney function and the use of renoprotective strategies. Medications such as statins, NSAIDs, and corticosteroids are being investigated for their potential to reduce systemic inflammation in HIV patients. Targeted therapies that block specific cytokines or their receptors, such as monoclonal antibodies against IL-6 or TNF-α, have shown promise in reducing inflammation. Regular exercise, a healthy diet, and smoking cessation can help reduce inflammation and improve overall health. Effective treatment of co-infections can reduce the inflammatory burden and improve immune function. Regular monitoring of inflammatory markers such as CRP, IL-6, and TNF-α is essential for assessing the level of inflammation and guiding treatment decisions. Biomarkers can help identify patients at higher risk for inflammation-related comorbidities and allow for early intervention.^[19–21]^

### 7.2. Biomarkers for monitoring and therapeutic targets

Biomarkers play a critical role in the management of HIV by providing insights into disease progression, treatment efficacy, and the presence of comorbidities. In the context of chronic inflammation and immune activation, specific biomarkers can help identify patients at risk for complications and guide therapeutic interventions. Additionally, understanding these biomarkers can aid in the development of targeted therapies aimed at reducing inflammation and improving immune recovery. CRP is an acute-phase protein produced by the liver in response to inflammation. Elevated CRP levels are associated with an increased risk of cardiovascular disease, metabolic syndrome, and other inflammatory conditions in HIV patients. CRP is a widely used biomarker due to its stability and ease of measurement. IL-6 is a pro-inflammatory cytokine involved in immune regulation and inflammatory response. High levels of IL-6 are linked to persistent immune activation, cardiovascular risk, and neurocognitive decline in HIV patients. IL-6 can serve as a marker for systemic inflammation and an indicator of treatment efficacy. TNF-α is a central mediator of inflammation and immune activation. Elevated TNF-α levels are associated with immune system dysregulation, apoptosis of CD4^+^ T cells, and the maintenance of viral reservoirs. Monitoring TNF-α can help assess the inflammatory state and the effectiveness of anti-inflammatory treatments. sCD14 is a marker of monocyte activation and microbial translocation. Elevated sCD14 levels indicate increased immune activation and inflammation, and are associated with a higher risk of cardiovascular disease and mortality in HIV patients. sCD14 can provide insights into gut integrity and systemic immune activation. D-dimer is a fibrin degradation product that is elevated in states of hypercoagulability and inflammation. High D-dimer levels are associated with an increased risk of thromboembolic events and cardiovascular disease in HIV patients. Monitoring D-dimer can help assess coagulation status and inflammation.^[22,23]^

The absolute count of CD4^+^ T cells is a primary indicator of immune system health and recovery in HIV patients. Increases in CD4^+^ T cell counts after ART initiation reflect successful suppression of viral replication and immune restoration. The ratio of CD4^+^ To CD8^+^ T cells provide additional information about immune recovery. A normalizing CD4/CD8 ratio indicates balanced immune reconstitution, while a persistently low ratio suggests ongoing immune dysregulation. IL-2 is critical for T cell proliferation and survival. Monitoring IL-2 levels can provide insights into the regenerative capacity of the immune system and the effectiveness of immune-based therapies. Monoclonal antibodies against IL-6 or its receptor (e.g., tocilizumab) have shown promise in reducing inflammation and improving clinical outcomes in inflammatory diseases. These inhibitors may help mitigate the effects of chronic inflammation in HIV patients. Anti-TNF-α therapies (e.g., infliximab, adalimumab) can reduce systemic inflammation and improve immune function. These agents are widely used in autoimmune diseases and could be repurposed for managing HIV-associated inflammation. Beyond their lipid-lowering effects, statins possess anti-inflammatory properties. They can reduce CRP levels and improve endothelial function, potentially lowering cardiovascular risk in HIV patients. NSAIDs like ibuprofen and naproxen can reduce inflammation by inhibiting cyclooxygenase enzymes. While useful for short-term management, their long-term use requires careful monitoring due to potential side effects. Modulating the gut microbiome with probiotics and prebiotics can improve gut barrier function and reduce microbial translocation, thereby lowering systemic inflammation. Agents like N-acetylcysteine and vitamin E can counteract oxidative stress and inflammation. Their use in HIV patients may help reduce the inflammatory burden and support immune recovery. The use of biomarkers allows for personalized treatment approaches in HIV management. Regular assessment of inflammatory markers and immune recovery indicators can guide therapeutic decisions and adjustments. Patients with elevated inflammatory markers may benefit from adjunctive anti-inflammatory therapies, while those with poor immune recovery might require additional interventions to support CD4^+^ T cell regeneration.^[21–23]^

### 7.3. Role of ART in HIV treatment

ART represents a cornerstone in the treatment and management of HIV infection.^[24]^ ART comprises a combination of antiretroviral drugs that aim to suppress viral replication, preserve immune function, and ultimately, improve the quality of life and extend the lifespan of individuals living with HIV. ART works by inhibiting different stages of the HIV life cycle. By targeting viral enzymes crucial for replication, such as reverse transcriptase, protease, and integrase, ART effectively reduces the viral load in the bloodstream.^[25]^ Lowering viral replication rates helps control HIV infection and prevents the progression to acquired immunodeficiency syndrome (AIDS).^[26]^ By suppressing viral replication, ART allows the immune system to recover.^[27]^ It helps in restoring CD4^+^ T cell counts, which are vital for orchestrating immune responses. As a result, ART helps in strengthening the immune system, reducing susceptibility to opportunistic infections, and enhancing overall health.^[28]^

With effective viral suppression, ART significantly decreases the risk of developing HIV-related complications and opportunistic infections.^[29]^ It has led to a dramatic decline in AIDS-related deaths and has transformed HIV into a manageable chronic condition for many individuals. When adhered to consistently and effectively, ART can lower the amount of HIV virus in bodily fluids (such as blood, semen, vaginal fluids) to undetectable levels.^[30]^ This achievement of an undetectable viral load not only benefits the individual’s health but also greatly reduces the risk of HIV transmission to sexual partners, commonly referred to as “Undetectable = Untransmittable” (U = U). Despite the efficacy of ART, adherence to the prescribed regimen is crucial.^[31]^ Nonadherence can lead to the development of drug resistance, treatment failure, and the resurgence of viral replication.^[32]^ Additionally, some individuals may experience side effects from ART medications, which can affect adherence and long-term treatment success. Overall, Antiretroviral Therapy has been transformative in managing HIV infection. Early initiation of ART, coupled with sustained adherence, has the potential to significantly prolong the lifespan and improve the quality of life for individuals living with HIV, reducing HIV transmission rates, and moving closer to the goal of ending the HIV/AIDS epidemic.^[33]^

### 7.4. Influence of cytokines on recovery trajectory

The influence of cytokines on the recovery trajectory of HIV patients undergoing ART is a multifaceted aspect that significantly impacts treatment outcomes and immune reconstitution.^[34]^ Cytokines, as key signaling molecules in the immune system, orchestrate complex interactions that shape the response to HIV infection and subsequent recovery.^[35]^ Cytokines play a crucial role in restoring immune function in HIV-infected individuals receiving ART.^[36]^ Certain cytokines, such as interleukins (e.g., IL-7, IL-15) and growth factors, contribute to the regeneration and proliferation of immune cells, including CD4^+^ T cells. These cytokines aid in the rebuilding of the immune system, crucial for achieving immune recovery and reducing susceptibility to opportunistic infections. Dysregulated cytokine production, especially pro-inflammatory cytokines (e.g., IL-6, TNF-α), contributes to chronic immune activation and inflammation observed in HIV infection.^[37]^ Persistent immune activation, despite viral suppression by ART, can hinder immune recovery, negatively impact organ function, and lead to comorbidities. Cytokine profiles have been investigated as potential predictors of treatment outcomes in HIV patients on ART.^[38]^

Imbalances in cytokine networks influence the delicate equilibrium between immune activation and regulation.^[39]^ Shifts in cytokine profiles may contribute to immunopathogenesis, impacting the pace and extent of immune recovery. Modulating these imbalances could hold therapeutic potential in optimizing recovery trajectories. Some cytokines serve as biomarkers for monitoring disease progression and treatment responses in HIV patients.^[40]^ Monitoring changes in cytokine levels over time could offer insights into the effectiveness of ART and the likelihood of immune recovery. Additionally, targeting specific cytokines or their pathways could be explored as adjunctive therapies to enhance immune reconstitution. Variability in cytokine expression among individuals, differences in cytokine responses to ART, and challenges in standardizing cytokine measurements pose complexities in understanding their precise influence on the recovery trajectory.^[41]^ Furthermore, the interplay between cytokines, viral reservoirs, and host factors adds layers of complexity to predicting recovery outcomes accurately. Understanding the intricate roles of cytokines in immune modulation, inflammation, and immune recovery during ART is pivotal for optimizing treatment strategies, identifying potential therapeutic targets, and tailoring interventions aimed at improving recovery trajectories and long-term health outcomes for HIV-infected individuals.

## 8. Challenges and limitations

In the realm of studying cytokines and their influence on the recovery trajectory of HIV patients undergoing ART, several challenges and limitations persist, impacting research and clinical implications.^[42]^ Variability in cytokine expression among individuals, influenced by factors such as genetic predisposition, viral strain diversity, co-infections, and individual immune responses, complicates establishing uniform cytokine patterns predictive of recovery trajectories.^[41]^ Lack of standardized protocols for cytokine measurement techniques and assays across studies introduces variability and inconsistency in reported cytokine levels. This variability makes it challenging to compare findings and draw definitive conclusions.^[43]^ Cytokine profiles are highly dynamic, fluctuating in response to various factors including time of infection, stage of disease, treatment duration, and concurrent infections or comorbidities. Capturing the temporal changes in cytokine expression accurately poses a challenge.^[44]^ Cytokines interact in complex networks, exhibiting cross-regulation and pleiotropic effects.^[45]^ Isolating the impact of individual cytokines on recovery trajectories amid this intricate network of interactions remains challenging. Obtaining samples for cytokine analysis, such as blood or tissue samples, especially longitudinally, can be logistically challenging.^[46]^ Additionally, accessing specialized equipment and expertise for cytokine assays might not be universally available, limiting research in certain settings. Heterogeneity among HIV-infected populations, including differences in demographics, disease progression, comorbidities, and treatment adherence, can lead to diverse cytokine responses.^[47]^ This variability complicates the interpretation and generalizability of findings. Conducting longitudinal studies to track cytokine dynamics and recovery trajectories might pose ethical challenges, particularly in resource-limited settings or with vulnerable populations, raising issues of participant burden and access to healthcare.^[48]^ Establishing causation between specific cytokine profiles and recovery trajectories is challenging.^[49]^ Correlation between cytokine patterns and outcomes does not always imply causation, requiring cautious interpretation of study findings. Addressing these challenges requires concerted efforts to standardize methodologies, conduct longitudinal studies with diverse populations, employ advanced analytical techniques, and collaborate across research disciplines.^[50]^ Overcoming these limitations will enhance our understanding of cytokine influences on recovery trajectories, aiding in the development of targeted interventions and personalized treatment strategies for HIV patients on ART.

## 9. Future directions and implications

Exploring future directions and implications in understanding the influence of cytokines on the recovery trajectory of HIV patients undergoing ART offers promising avenues for research and clinical applications.^[20]^ Advancements in understanding individual variability in cytokine responses may pave the way for personalized treatment strategies.^[51]^ Tailoring ART regimens based on specific cytokine profiles or immune signatures could optimize therapeutic outcomes. Exploring cytokine modulation as a therapeutic approach in HIV management holds potential. Developing interventions targeting cytokine pathways to modulate immune responses, reduce chronic inflammation, and enhance immune recovery is an area of interest.^[52]^ Conducting comprehensive longitudinal studies to capture dynamic changes in cytokine profiles throughout different stages of HIV infection and ART is crucial. These studies can elucidate evolving patterns and their impact on recovery trajectories over time. Integrating omics approaches, such as genomics, transcriptomics, proteomics, and metabolomics, with cytokine analysis could provide a comprehensive understanding of the molecular mechanisms underlying cytokine-mediated recovery trajectories. Translating research findings into clinical practice necessitates developing interventions that leverage cytokine knowledge to optimize treatment outcomes. Clinical trials exploring cytokine-targeted therapies or immune modulators in conjunction with ART are warranted.^[53]^ Bridging the gap between research findings and implementation, especially in resource-limited settings, is crucial. Affordable and accessible methods for cytokine analysis and potential therapeutic interventions need to be developed to benefit diverse populations. Collaboration among immunologists, infectious disease specialists, clinicians, and bioinformaticians is essential. Multidisciplinary approaches can facilitate a deeper understanding of cytokine dynamics and their impact on HIV recovery trajectories. Educating healthcare providers and patients about the role of cytokines in HIV recovery trajectories can aid in informed decision-making, treatment adherence, and proactive management strategies.^[53]^ Ethical considerations regarding equitable access to cytokine-based diagnostics and therapies need to be addressed. Ensuring that benefits from advancements in cytokine research reach all populations is imperative.^[54–59]^ The future landscape in cytokine research for HIV recovery trajectories involves leveraging technological advancements, individualizing treatment approaches, developing targeted interventions, and translating scientific knowledge into tangible clinical benefits for improving outcomes and quality of life for HIV-infected individuals undergoing ART.

## 10. Implications for clinical and health policy making

The influence of cytokines on the recovery trajectory of HIV patients on ART has significant implications for both clinical practice and health policy making in several ways.^[60]^

### 10.1. Clinical implications

*Treatment monitoring:* Understanding cytokine profiles can help clinicians monitor the immune status and response to ART in HIV patients. Monitoring cytokine levels may aid in assessing the effectiveness of therapy and identifying individuals at higher risk of complications.^[61]^

*Individualized therapy:* Cytokine analysis may contribute to personalized medicine by guiding clinicians in tailoring treatment approaches. It could help identify patients who may benefit from additional interventions or modified treatment regimens based on their cytokine profiles.^[62]^

*Predicting disease progression:* Cytokine levels could serve as biomarkers for predicting disease progression or identifying patients at risk of treatment failure, allowing for proactive intervention strategies.^[63]^

*Management of co-infections and comorbidities:* Cytokine research might reveal insights into managing co-infections or comorbidities commonly associated with HIV. Understanding cytokine interactions could inform strategies for managing conditions like tuberculosis or cardiovascular diseases in HIV-positive individuals.^[64]^

### 10.2. Health policy implications

*Resource allocation:* Health policies influenced by cytokine research might emphasize the allocation of resources towards cytokine profiling, enabling better monitoring of HIV patients. This could involve funding for specialized testing, research, and training for healthcare professionals.

*Guideline development:* Cytokine data can contribute to the development or modification of clinical guidelines for HIV management. Policy makers might incorporate recommendations based on cytokine profiles into national or international treatment protocols.

*Research funding and prioritization:* Health policies may prioritize funding for research into cytokines and their role in HIV pathogenesis and treatment. Government agencies and institutions might allocate resources to encourage studies exploring cytokine modulation and its impact on patient outcomes.

*Access to advanced diagnostic tools:* Policies can support wider access to advanced diagnostic tools capable of assessing cytokine levels. This might involve negotiations with pharmaceutical companies or subsidizing costs to make these tests more accessible in healthcare settings.

*Education and training:* Health policies might emphasize educational programs for healthcare providers to interpret cytokine data effectively. This could involve integrating cytokine analysis into medical training curriculum or offering continuing education opportunities.

## 11. Conclusion

The management of HIV has significantly evolved with the advent of ART, transforming it into a chronic manageable condition. However, persistent immune activation and chronic inflammation remain significant challenges, contributing to a range of comorbidities such as cardiovascular disease, neurocognitive disorders, metabolic syndrome, bone disorders, and renal disease. Pre-ART cytokine dysregulation lays the foundation for chronic inflammation, with elevated levels of pro-inflammatory cytokines driving immune system activation and damage. Initiating ART reduces viral replication and partially alleviates inflammation, but residual immune activation often persists, underscoring the need for additional therapeutic strategies. Successful CD4^+^ T cell recovery, a primary goal of ART, is influenced by several factors including baseline CD4^+^ counts, age, co-infections, and the timing of ART initiation.

Persistent immune activation, even in the context of ART, is driven by factors such as microbial translocation, residual viral replication, and co-infections. This chronic inflammatory state is marked by elevated levels of cytokines like IL-6, TNF-α, and sCD14, which are associated with various comorbidities. Addressing chronic inflammation is essential to reduce the risk of these conditions and improve the overall quality of life for HIV patients. Monitoring inflammatory biomarkers such as CRP, IL-6, TNF-α, and sCD14, alongside immune recovery markers like CD4^+^ T cell count and the CD4/CD8 ratio, provides valuable insights into the inflammatory state and immune health of HIV patients. These biomarkers not only guide treatment decisions but also help in assessing the effectiveness of therapeutic interventions aimed at reducing inflammation and enhancing immune recovery.

## Author contributions

**Conceptualization:** Emmanuel Ifeanyi Obeagu.

**Methodology:** Emmanuel Ifeanyi Obeagu.

**Supervision:** Emmanuel Ifeanyi Obeagu.

**Visualization:** Emmanuel Ifeanyi Obeagu.

**Writing – original draft:** Emmanuel Ifeanyi Obeagu.

**Writing – review & editing:** Emmanuel Ifeanyi Obeagu.
